# Multiple Intravenous Injections of Valproic Acid-Induced Mesenchymal Stem Cell from Human-Induced Pluripotent Stem Cells Improved Cardiac Function in an Acute Myocardial Infarction Rat Model

**DOI:** 10.1155/2020/2863501

**Published:** 2020-12-17

**Authors:** Shuyuan Guo, Yusen Zhang, Yanmin Zhang, Fanhua Meng, Minghua Li, Zhendong Yu, Yun Chen, Guanghui Cui

**Affiliations:** ^1^Shantou University, School of Medicine, China; ^2^Peking University Shenzhen Hospital, Ultrasound Department, China; ^3^Peking University Shenzhen Hospital, Central Laboratory, China; ^4^Peking University Shenzhen Hospital, Reproductive Medical Center, China; ^5^Shenzhen Key Laboratory of Drug Addiction and Safe Medication, China

## Abstract

Mounting evidence indicates that the mesenchymal stem cell (MSC) injection is safe and efficacious for treating cardiomyopathy; however, there is limited information relating to multiple intravenous injections of human-induced pluripotent stem cell-derived mesenchymal stem cell (hiPSC-MSC) and long-term evaluation of the cardiac function. In the current study, MSC-like cells were derived from human-induced pluripotent stem cells through valproic acid (VPA) induction and continuous cell passages. The derived spindle-like cells expressed MSC-related markers, secreted angiogenic and immune-regulatory factors, and could be induced to experience chondrogenic and adipogenic differentiation. During the induction process, expression of epithelial-to-mesenchymal transition- (EMT-) related gene N-cadherin and vimentin was upregulated to a very high level, and the expression of pluripotency-related genes Sox2 and Oct4 was downregulated or remained unchanged, indicating that VPA initiated EMT by upregulating the expression of EMT promoting genes and downregulating that of pluripotency-related genes. Two and four intravenous hiPSC-MSC injections (10^6^ cells/per injections) were provided, respectively, to model rats one week after acute myocardial infarction (AMI). Cardiac function parameters were dynamically monitored during a 12-week period. Two and four cell injections significantly the improved left ventricular ejection fraction and left ventricular fractional shortening; four-injection markedly stimulated angiogenesis reduced the scar size and cell apoptosis number in the scar area in comparison with that of the untreated control model rats. Although the difference was insignificant, the hiPSC-MSC administration delayed the increase of left ventricular end-diastolic dimension to different extents compared with that of the PBS-injection control. No perceptible immune reaction symptom or hiPSC-MSC-induced tumour formation was found over 12 weeks. Compared with the PBS-injection control, four injections produced better outcome than two injections; as a result, at least four rounds of MSC injections were suggested for AMI treatment.

## 1. Introduction

Coronary artery disease is a major cause of morbidity and mortality worldwide. Despite recent achievements in acute myocardial infarction (AMI) treatment, the irreversible damage to cardiomyocytes after AMI leads to left ventricular (LV) remodeling and ischemic heart failure [1, 2].

Mesenchymal stem cells (MSCs) are mesoderm-derived cells with the capacity to differentiate into multicell lineages. MSCs exert an immune tolerant phenotype which is characterized by low level expression of MHC antigens and the lack of costimulatory molecules. Through releasing soluble immune modulators such as indoleamine 2, 3 dioxygenase (IDO), prostaglandin E2 (PGE-2), or nitric oxide (NO), MSCs suppress activation of immune cells [3].

In recent years, mesenchymal stem cell transplantation has emerged as a potential treatment means for ischemic heart disease [4–6]. In clinical trials, intracoronary delivery of bone marrow mononuclear cells or bone marrow MSCs has provided evidence of improved cardiac function, reduced scar area, and reduced prevalence of recurrent MI, stent thrombosis, and death [7–11]. Accumulating evidence has suggested that following AMI, chronic, excessive proinflammatory immune responses may account for progressive adverse remodeling and dysfunction of the myocardium, and the transplanted MSCs exert its therapeutic benefits by secreting factors to stimulate local angiogenesis and alleviate inflammatory activities [12–15].

To date, most experimental and clinical studies have used an intramyocardium or intracoronary delivery system. Intravenous cell delivery is advantageous for AMI treatment at a practical level, and multiple rounds of intravenous injections of MSCs are convenient and tolerable from the viewpoint of patients, whereas repeated intromyocardium or introcoronary cell delivery is not. However, numerous reports indicate that only a small portion of the injected cells could integrate into the infarct area after intravenous injections, while most of them are trapped in the lung, kidney, liver, and spleen, and are gradually cleared away in a period of time [16–18]. Even with the application of molecule targeting delivery systems, such as ICAM or SDF1/CXCR4, the retaining rate of the injected cells in the infracted area is still less than 30% [19, 20].

Ne´ meth et al. demonstrated that the exogenous MSCs accumulated in the lung could transmigrate outside to the vascular space and rapidly interact with lung-resident tissue macrophages; stimulated by the MSCs, endogenous macrophages produce large amount of interleukin-10. Since this seminal report, there has been a plurality of reports validating a unique cross-talk between exogenous MSCs and recipient monocyte and/or macrophages as part of the anti-inflammatory effect of MSCs [21]. Completely curbing excessive and prolonged inflammation in myocardium by a single intravenous administration of MSCs is impossible; therefore, repeated infusions over time might exert superior, prolonged anti-inflammatory effects.

Despite the advantages of using MSCs derived from the bone marrow, adipose tissue, or umbilical cord for cell therapy, several issues incurred by properties of these cells limit their application in clinical use. For example, they are limited in number and quickly lose their differentiation potential during in vitro expansion [22]. As for autologous application, MSCs decline in quantity and quality with age, isolated MSCs are often composed of heterogeneous cell populations, and each population may have different proliferative and differentiation potential that make clinic application difficult [23, 24]. A large number of primitive MSCs can be isolated from the perinatal tissue, but it can be obtained from only around one-third of umbilical cord blood specimens, and the access to the abortal tissue requires ethical approval [25, 26].

The human induced pluripotent stem cell (hiPSC) is an attractive stem cell source for cell therapy. In comparison to isolation of primary cells, iPSCs can be harvested free of ethical constraints and offer a unified starting point for generation of standardised derivatives at large scale [27]. Thus, differentiating hiPSC into MSC (hiPSC-MSC) before transplantation is one of the most promising approaches for the safe and effective use of the induced pluripotent stem cells.

Before clinic application of the induced hiPSC-MSC, there are questions remaining to be answered. First, do the intravenous injected hiPSC-MSCs have tumor formation potential in the hosts? Second, will immune reaction to transplanted cells be initiated and accelerated by repeated injections of hiPSC-MSCs? Third, what is the optimal cell dosage for improving the cardiac function without causing fatal side effects?

Valproic acid (VPA), a histone deacetylase inhibitor (HDI), is used as a mood stabiliser and antiepileptic drug. Recently, it has been found that VPA treatment could modulate multipotency, migration, proliferation, and differentiation of cord blood and periodontal MSCs [28, 29].

Epithelial-to-mesenchymal transition (EMT) has been regarded as the first stage of mesoderm commitment in hESCs [30], and VPA plays an important role in the process of cell mesoderm commitment induction [31].

In this study, we hypothesised that MSC-like cells could be derived from human hiPSCs by VPA induction and planned to undertake two and four rounds of intravenous administration of the derived hiPSC-MSCs in a rat model of AMI. By doing so, we attempted to test the safety and the effectiveness of the derived MSC-like cells in treating AMI within a 12-week period.

## 2. Materials and Methods

### 2.1. Human-Induced Pluripotent Stem Cell Cultures

Human iPSC cell line PBMC-5 (derived from the peripheral blood mononuclear cell of a 31-year-old Chinese Han female) was kindly provided by Dr Tiancheng Zhou at the Guangzhou Institute of Biomedicine & Health, Chinese Academy of Sciences, under the appropriate human ethics and material transfer agreements. hiPSCs were routinely grown as bulk cultures on a widely used commercially available defined medium, mTeSR1 (Stem Cell Technologies, Vancouver, BC, Canada), in combination with the cell attachment matrix Matrigel (BD Biosciences, San Diego, USA).

### 2.2. MSC Differentiation of hiPSC

hiPSC bulk cultures were seeded into the mTeSR1 medium on a Matrigel-coated six-well plate as large colonies at high confluence, and then the medium was changed to the MSC differentiation medium containing DMEM-Ham's F-12 basal medium supplemented with 20% knockout serum replacement (KOSR), 1 mM L-glutamine, 10 mM nonessential amino acids, 50 U/mL penicillin/streptomycin (Invitrogen, Carlsbad, CA, USA), and 0.5 mM VPA (Sigma-Aldrich, St. Louis, USA). The MSC differentiation medium was replaced every other day for eight days, and then the cells were passaged to a single cell suspension using the TrypleSelect (Invitrogen). Single cells were seeded at a density of 40,000 cells per cm^2^ into the MSC medium (DMEM high glucose basal medium supplemented with 10% foetal bovine serum, 1 mM L glutamine, 50 U/mL penicillin/streptomycin, and 10 mM nonessential amino acids, all from Invitrogen) as the first passage, and then in the subsequent passages, 20,000 cells per cm^2^ were seeded.

### 2.3. Quantitative Real-Time RT-PCR

Total RNA was isolated using the RNase mini kit (Qiagen, Valencia, CA, USA). A high-capacity RNA-to-cDNA kit (Applied Biosystems, Foster City, CA, USA) was used to reverse transcribe 1 *μ*g of RNA into cDNA with MMLV reverse transcriptase and random primers according to the manufacturer's instructions (85°C, 5 s, and 37°C, 1 h). Real-time PCR was performed using specific primers under optimal reaction conditions (94°C, 30 s; 60°C, 35 s; 72°C, 40 s; 30 cycles) for quantification of the gene expression using SYBR-Green RT-PCR reagents (Applied Biosystems). The 2-*ΔΔ*Cq method was used to analyze relative gene expression level.  Table 1 lists the primers used.

### 2.4. Detection of MSC Specific Markers by Flow Cytometry

Adherent spindle-like cells at the sixth passage were harvested by trypsinization. They were resuspended for measuring expression of typical MSC surface markers (CD29, CD44, CD73, CD90, and CD105) as well as markers of hematopoietic cell lineages (CD34, CD45, and human leukocyte antigen-DR isotype) by fluorescent-activated cell sorting (FACS). Fluorescent conjugated antibodies: anti-CD29, CD73, CD90, and CD105 (Biolegend, San Diego, CA, USA); anti-CD45, CD34, CD44 and HLA-DR (Boster, Wuhan, China). Isotype control antibodies were used as negative controls to optimise parameters during analyses using BD-FACScan (Becton Dickinson, Franklin Lakes, NJ, USA). Data were analyzed using FlowJo (Ashland, OR, USA).

### 2.5. Differentiation Assay of hiPSC-MSC

Induction of chondrogenic and adipogenic differentiation and subsequent staining of induced cells were conducted as described previously [32]. Chondrogenic differentiation was induced by culturing cells with a chondrogenic induction medium (DMEM supplemented with 10% FBS, 6.25 *μ*g/mL of insulin, 50 nM of ascorbate phosphate, and 10 ng/mL of transforming growth factor-*β*) for two weeks. Formation of sulphated proteoglycans was visualised by Alcian Blue staining.

To induce adipocytic differentiation, cells were cultured as monolayers, allowing to become near confluent and then maintained in the adipogenic induction medium (DMEM supplemented with 10% FBS, 10^−7^ M of dexamethasone, and 10 *μ*g/mL of insulin) for two weeks. Then, cells were assessed by Oil-Red-O staining as an indicator of intracellular lipid accumulation.

### 2.6. Cytokine Production Analysis

Two million umbilical cord derived MSCs (UC-MSCs) and hiPSC-MSCs were, respectively, seeded in a 10 cm diameter culture dish in the MSC medium for the purpose of comparing the cytokine production level between these two cell groups. After a three-day culture period, the supernatants were collected for detecting IGF, VEGF, HGF, IDO, PGE2, and IL-10 production. Cytokine concentration was measured using ELISA detection kits according to the manufacturer's instructions (IGF and PGE2, Multi Science, Hangzhou, China; VEGF and HGF, Abcam, Cambridge, MA, USA; IDO, RayBio Norcross, GA, USA; IL-10, Sino Biological, Beijing, China). Samples were analyzed using a 1510 spectrophotometer (Thermo Fisher, Vantaa, Finland).

### 2.7. Animals and AMI Induction

Male Sprague–Dawley rats (4 weeks, body mass 230-270 g) were purchased from the Medical Experimental Animal Centre of Guangdong Province (Guangdong, China). They were housed in a room at constant temperature (20°C) and humidity (45%) on a 12-h light–dark cycle. Rats had free access to standard laboratory chow and tap water.

Approval for animal experimentation was obtained from the Ethics Committee of Peking University Shenzhen Hospital. Chinese laws relating to the care and treatment of animals were followed strictly throughout the study and complied with Animal Research: Reporting of In Vivo Experiments (ARRIVE) guidelines.

A left thoracotomy was undertaken between the fourth and fifth ribs of rats. A suture was constructed around the left anterior descending coronary artery (LADCA). Ischemia was maintained for 40 min, followed by reperfusion. In sham control rats, the constructed suture was immediately removed to restore blood flow.

### 2.8. Evaluation of the Cardiac Function by Echocardiography

The cardiac function was evaluated using a two-dimensional echocardiography system (Vevo 2100; Visual Sonics, Toronto, ON, Canada) with an MS250 transducer (13–24 MHz). We measured parameter changes pertaining to the left ventricular ejection fraction (LVEF), left ventricular fractional shortening (LVFS), and left ventricular end-diastolic dimension (LVEDD).

### 2.9. Intravenous Administration of hiPSC-MSC in the Rat Model of AMI

One week after AMI, model rats were anaesthetised (40 mg/kg body mass of pentobarbital sodium), and the cardiac function was evaluated by echocardiography. Rats with LVEF lower than 55% were selected for this experiment and divided randomly into two hiPSC-MSC injection groups (two and four injections, *n* = 8) and one phosphate-buffered saline (PBS) injection control group (*n* = 8). Two and four intravenous boli of 1 × 10^6^ hiPSC-MSCs (passage 6) were given, respectively, to rats in the hiPSC-MSC-treated groups, and PBS was provided to rats in the control group. hiPSC-MSC intravenous injections were carried out once a week. The cardiac function was evaluated once every two weeks after the first infusion in the 12-week period.

### 2.10. Quantification of the Scar Size

Masson's Trichrome staining was used to assess the area of myocardial scars 12 weeks after AMI induction. This procedure stains the viable area pink and the scar area blue. Briefly, three equal slices below the suture were fixed in 4% formaldehyde and embedded in paraffin and cut into sections of thickness 5 *μ*m. To detect fibrosis, the sections were stained with Masson's Trichrome and assessed by a pathologist blind as to study protocol. The percentage of the scar area was quantified by a computerised morphometry system with a microscope (Olympus, Tokyo, Japan).

### 2.11. Vascularisation Evaluation in the Scar Area

Three frozen equal slices below the suture were fixed and blocked for immunohistochemical detection of the *α*-SMA expression. Primary mouse anti-rat *α*-SMA antibodies (Biolegend, San Diego, CA, USA), secondary biotin-coupled anti-mouse IgG antibody, and avidin-horseradish peroxidase conjugates (Boster, Wuhan, Hubei, China) were applied, respectively, following a standard immunohistochemistry procedure. Irrelevant antibody was used as a negative control. *α*-SMA^+^ cell capillary in the scar regions was counted by a high-power field (200×) in each section, and at least five randomly fields in each section were analyzed.

### 2.12. Cell Apoptosis Assay

The TdT-mediated dUTP nick-end labeling (TUNEL) assay was performed on heart sections according to the manufacturer's recommendations (KeyGEN BioTECH, Nanjing, Jiangsu, China) to quantify cardiomyocyte apoptosis. Counterstaining with hematoxylin was conducted to visualize normal nuclei. Apoptotic cells were counted in 5 randomly selected high-power fields (400×) from the border and infarcted area of three sections from each heart and averaged.

### 2.13. Statistical Analyses

Data were shown as mean ± SD and analyzed using SPSS software, Version 11.0. One-way ANOVA with LSD*-t* was used in multiple group comparisons, and *P* < 0.05 was considered significant.

## 3. Results

### 3.1. Morphology Transformation during hiPSC-MSC Induction

**Figure 1:**
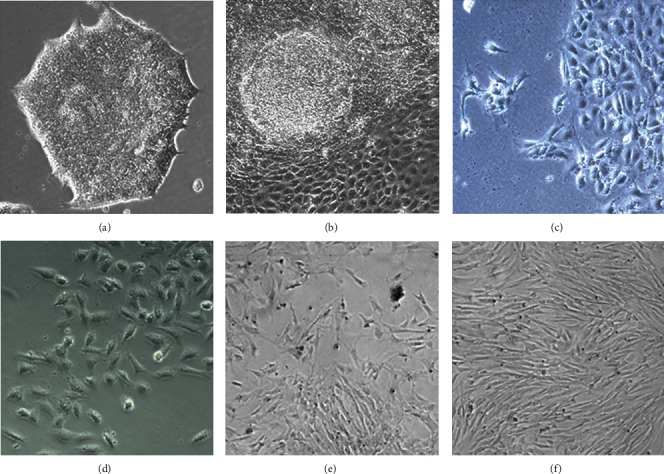
Morphology transformation during hiPSC-MSC induction. (a) Undifferentiated human iPSC colony. (b) hiPSCs transformed to larger cuboidal-shaped epithelial-like cells after eight-day VPA induction. (c) After the first passage, the morphology of differentiating cells transformed from cuboidal to olive shape. (d) Large spindle-shaped cells appeared in the second passage. (e) Small spindle-shaped cells grew out from cell mess. (f) On the passage six, more than 90% of the adherent cells maintained a small spindle-shaped morphology 100×.

Without the presence of VPA, undifferentiated hiPSCs cultured on Matrigel in the mTeSR1 medium appeared as dense, tightly packed cell colonies. Eight-day incubation with VPA drove more than 60% of hiPSCs to form a monolayer of larger cuboidal-shaped epithelial-like cells, leaving only small islands of undifferentiated cells. From the second passage in the MSC medium, the morphology of differentiating cells gradually altered from larger cuboidal-shaped epithelial-like towards olive shape-like, and then on the sixth passage, more than 90% of the adherent cells maintained a small spindle-shaped morphology (Figure 1).

### 3.2. VPA Promoted the EMT-Related Gene Expression

**Figure 2:**
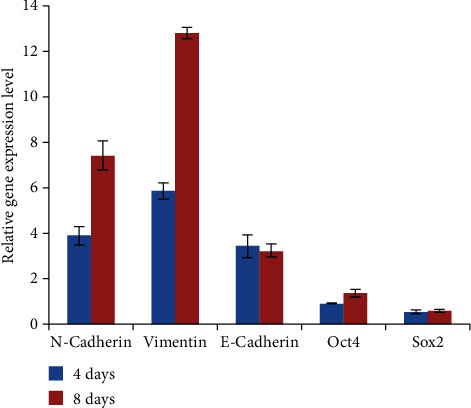
VPA treatment up-regulated EMT-related gene expression.

To examine whether, or not, VPA could induce hiPSCs to mesoderm commitment, pluripotency-related transcription factors and EMT-related gene expression were evaluated at the fourth and eighth induction days. In comparison with DMSO-treated control hiPSCs, four-day VPA induction significantly upregulated the expression of the EMT-related gene N-cadherin and vimentin, and the upregulation was continued to day eight. The pluripotency-related transcription factor Sox2 was downregulated, while the Oct4 expression remained at a similar level. Unexpectedly, the EMT reverse process MET-related gene E-cadherin expression was upregulated (Figure 2).

In comparison with DMSO-treated control hiPSCs, four- to eight-day VPA treatment significantly upregulated the expression of the EMT-related gene N-cadherin and vimentin, and the pluripotency-related transcription factor Sox2 was downregulated, while the Oct4 expression remained at a similar level. The EMT reverse process MET-related E-cadherin gene expression was upregulated at the same time.

### 3.3. Differentiation Induction and Surface Marker Expression of hiPSC-MSC

**Figure 3:**
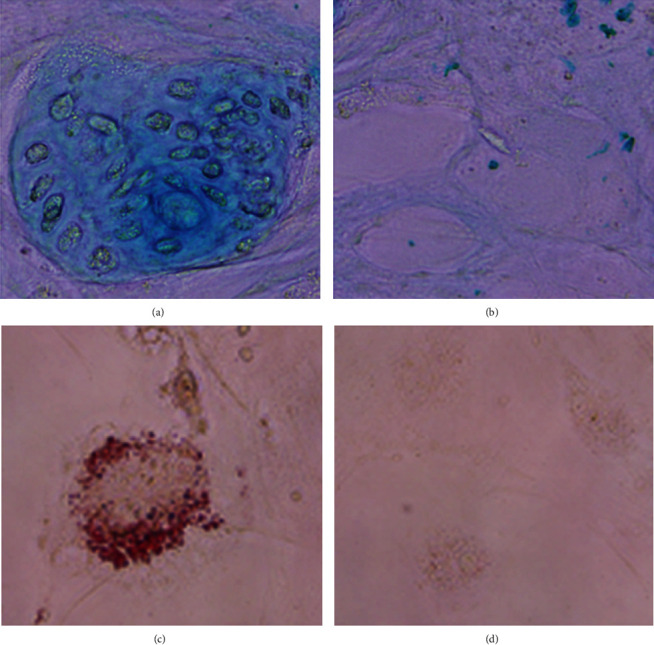
hiPSC-MSC chondrogenic and adipogenic differentiation induction. (a) Alcian Blue staining for sulphated proteoglycans. (b) Negative control. (c) Oil-Red-O staining for intercellular lipid vacuoles. (d) Negative control 400×.

**Figure 4:**
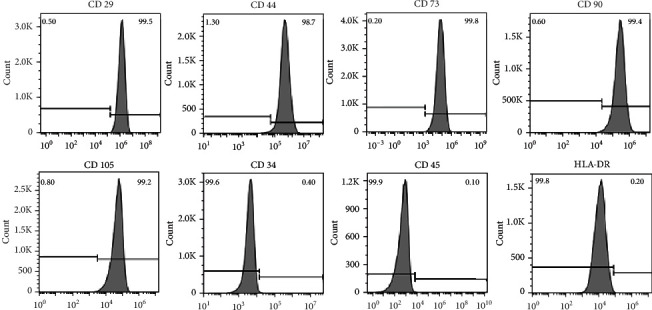
Surface marker expression detection of hiPSC-MSC.

Cells at passage 6 were cultured with the chondrogenic differentiation medium for two weeks, and sulphated proteoglycans produced by cell plaques could be detected using Alcian Blue staining. When cells were cultured in the adipogenic induction medium, intercellular lipid vacuoles could be seen since day 10 by Oil-Red-O staining (Figure 3). More than 98% of passage 6 hiPSC-MSCs expressed typical MSC surface markers (CD29, CD44, CD73, CD90, and CD105), while the expression of markers of hematopoietic cell lineages (CD34, CD45, and HLA-DR) was below 0.4% (Figure 4).

More than 98% of passage 6 hiPSC-MSCs expressed typical MSC surface markers (CD29, CD44, CD73, CD90, and CD105), while the expression of markers of hematopoietic cell lineages (CD34, CD45, and HLA-DR) was below 0.4%.

### 3.4. hiPSC-MSC Produced Angiogenesis and Immune Regulatory Factors

**Figure 5:**
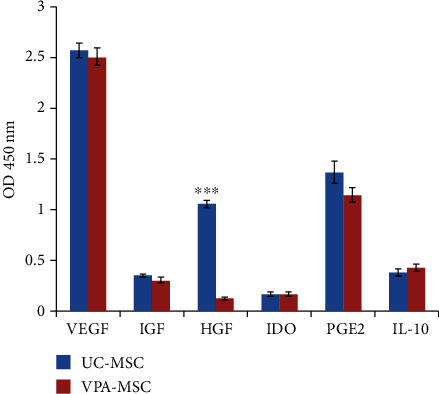
hiPSC-MSC produced angiogenic and immune regulatory factors.

Angiogenic and immune regulatory factor secretion level of hiPSC-MSCs was compared with that of UC-MSCs. UC-MSCs expressed IGF, VEGF, HGF, IDO, PGE2, and IL-10. The expression level of IGF, VEGF, IDO, PGE2, and IL-10 of hiPSC-MSCs did not significantly differ from that of UC-MSCs, but the HGF expression level of hiPSC-MSCs was significantly lower compared with that of UC-MSCs, *P* < 0.001 (Figure 5).

The expression level of VEGF, IGF, IDO, PGE2, and IL-10 of hiPSC-MSCs was not significantly different from that of UC-MSCs; however, the HGF expression level of hiPSC-MSCs was significantly lower compared with that of UC-MSCs, ^∗∗∗^*P* < 0.001.

### 3.5. hiPSC-MSC Dosages and Adverse Effects

In our preliminary experiment, severe dyspnea was found in some rats immediately after intravenous infusions of 2–3 × 10^6^ hiPSC-MSCs, and these rats died quickly (>30-50%), perhaps due to thromboemboli in the lungs formed by hiPSC-MSCs, as reported by other researchers [16, 17, 33]. As a result, we reduced the dose of each injection to 1 × 10^6^ cells, and used cells within six passages, to ensure that cells retained their small, spindle-like morphology, thereby facilitating their passage through small blood vessels in the lungs.

MSCs are immune-privileged and can be transplanted into unrelated recipients [3]. In order to test the immune tolerant and immunosuppressive effects of the induced hiPSC-MSCs, we used immune competent rats in this study. During the four rounds of infusions of 1 × 10^6^ hiPSC-MSCs to model rats and the whole 12-week period, symptoms relating to acute or chronic immunoreactions, such as dyspnea, convulsion, oedema, skin rash, langour, or progressive emaciation, were monitored: no perceptible symptoms were found. At the end of the twelfth week, anatomical examination of the model rats was undertaken, and no tumor formation or evident pathological changes were identified.

### 3.6. hiPSC-MSC Administrations Improved the Cardiac Function

**Figure 6:**
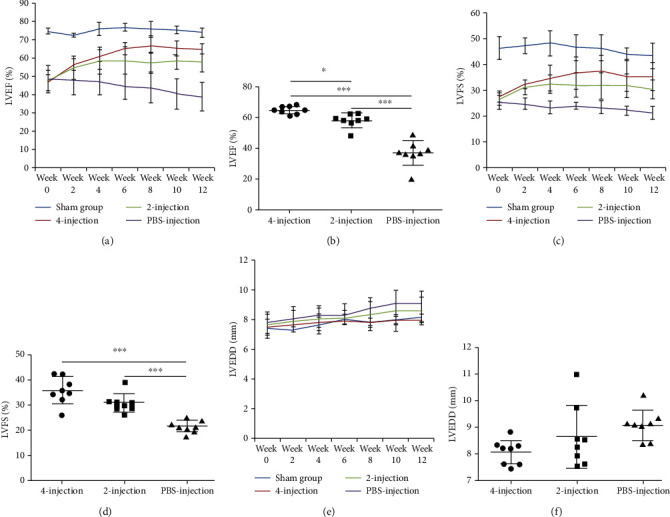
hiPSC-MSC administration improved the cardiac function. (a) LVEF of the PBS-injection group continued to decline from week 0 to week 12. hiPSC-MSC injection increased LVEF to different extents in cell-treated groups. (b) The improvement in LVEF in the hiPSC-MSC-injection groups was significant compared with that in the PBS-injection group at week 12 (PBS-injection group *v*. two-injection group, ^∗∗∗^*P* < 0.001; PBS-injection group *v*. four-injection group, ^∗∗∗^*P* < 0.001). There was a significant difference between the two-injection and the four-injection groups, ^∗^*P* < 0.05. (c) LVFS of the PBS-injection group kept declining from week 0 to week 12. hiPSC-MSC treatments increased LVFS to different levels in the MSC-injection groups. (d) Significant differences were found when comparing the improvement extent of LVFS of the hiPSC-MSC-injection groups with that of the PBS-injection group at the end of week 12 (PBS-injection group v. two-injection group, ^∗∗∗^*P* < 0.001; PBS-injection group v. four-injection group, ^∗∗∗^*P* < 0.001). (e) In the PBS-injection group, LVEDD increased continuously over 12 weeks. hiPSC-MSC treatments prevented the increase of LVEDD in the hiPSC-MSC-injection groups to different levels compared with that of the PBS-injection group. (f) The differences of LVEDD prevention among the hiPSC-MSC-injection groups and the PBS-injection group were insignificant at the end of 12 weeks.

Cardiac function improvement was analyzed by monitoring LVEF obtained at week 0 (the day on which the first hiPSC-MSC injection was given) and weeks 2, 4, 6, 8, 10, and 12. From week 0 to week 12 before sacrificing the rats, LVEF of the sham group kept unchanged; in the PBS-injection group, LVEF continued to decline, from 48.36 ± 7.30% to 36.94 ± 8.31%; in the two-injection group, LVEF increased from 47.44 ± 7.6% at week 0 to 57.9 ± 4.8% at week 12; in the four-injection group, LVEF grew from 46.67 ± 4.4% at week 0 to 64.7 ± 2.4% at week 12 (Figure 6(a)). Significant differences were found when comparing MSC-injection groups with the PBS-injection group at the end of 12 weeks (PBS-injection group v. two-injection group, *P* < 0.001; PBS group v. four-injection group, *P* < 0.001). There was a significant difference between the two-injection group and the four-injection group, *P* < 0.05 (Figure 6(b) and Table 2).

LVFS is an important indicator for evaluating the LV systolic function. From week 0 to week 12, LVFS of the sham group kept stable; in the PBS-injection group, LVFS declined from 25.12 ± 2.53% at week 0 to 19.75 ± 3.8% at week 12; in the two-injection group, LVFS increased from 27.64 ± 2.34% at week 0 to 30.67 ± 3.7% at week 12; in the four-injection group, LVFS ascended from 27.97 ± 2.34% at week 0 to 34.25 ± 5.4% at week 12 (Figure 6(c)). Significant differences were observed upon comparison of the PBS-injection group with the MSC-injection groups at week 12 (PBS-injection group v. two-injection group, *P* < 0.001; PBS-injection group v. four-injection group, *P* < 0.001), and there was no significant difference between the four-injection group and the two-injection group (Figure 6(d) and Table 3).

LVEDD was measured continuously after two to four rounds of hiPSC-MSC infusions over 12 weeks. In the sham group, no significant variation of LVEDD was found; in the PBS-injection group, LVEDD continued to increase from 7.8 ± 0.71 mm at week 0 to 9.36 ± 0.77 mm at week 12. In the MSC-injection groups, cell treatment prevented LVEDD increase to different extents from week 0 to week 12 compared with the PBS-injection group (two-injection group: from 7.65 ± 0.69 mm to 8.67 ± 0.86 mm; four-injection group: from 7.5 ± 0.52 mm to 8.02 ± 0.34 mm) (Figure 6(e)), despite the insignificant differences among these groups (Figure 6(f) and Table 4).

### 3.7. hiPSC-MSC Administration Reduced the Scar Size

**Figure 7:**
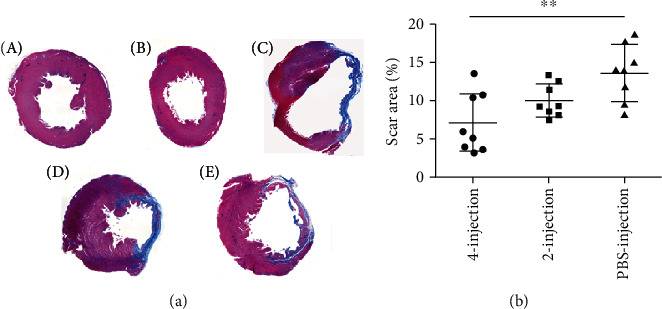
hiPSC-MSC administration reduced the scar area. (a) Masson's Trichrome staining, scar tissue was stained blue, and the viable area was pink. A. Normal heart. B. Sham infarcted heart. C. PBS-injection-treated AMI heart. D. Two-injection-treated AMI heart. E. Four-injection-treated AMI heart. (b) MSC treatments reduced the scar area in the cell-injection groups compared with those in the PBS-injection group, and there was significant difference in the four-injection group, ^∗∗^*P* < 0.01).

To analyze the potential effect of hiPSC-MSC therapy on the scar size in the AMI model, we stained sections of the scar area with Masson's trichrome. Twelve weeks after AMI, histology showed that scar tissue formation in the infarct area was composed mainly of the fibrotic tissue with some inflammatory cells, and the scar tissue was stained blue, and the viable area was pink (Figure 7(a)): PBS-injection group, 13.74 ± 3.4%; two-injection group, 10.18 ± 2.16%; and four-injection group, 7.08 ± 3.68%. MSC treatments reduced the scar area in the cell-injection groups compared with those in the PBS-injection group, and there was a significant difference in the four-injection group, *P* < 0.01 (Figure 7(b) and Table 5).

### 3.8. hiPSC-MSC Administrations Promoted Vascularisation in the Scar Area

**Figure 8:**
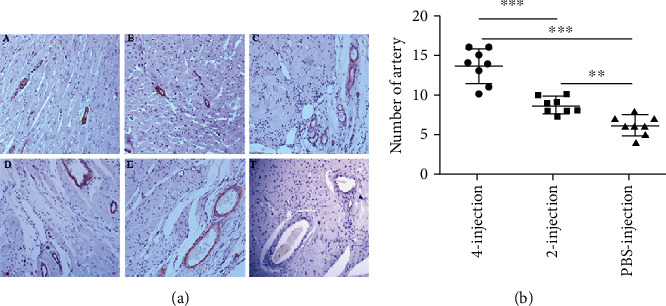
hiPSC-MSC administration stimulated angiogenesis in the scar area. (a) Detection of blood vessel by anti-*α*-SMA immunohistochemistry staining. A *α*-SMA^+^ blood vessels in a section of the normal rat (no scar area). B *α*-SMA^+^ blood vessels in a section of the sham operated rat (no scar area). C *α*-SMA^+^ blood vessels in a section of the four-injection-treated rat (in scar area). D *α*-SMA^+^ blood vessels in a section of the two-injection-treated rat (in scar area). E *α*-SMA^+^ blood vessels in a section the of PBS-injection-treated rat (in scar area). F Negative staining control, 200×. (b) The number/hpf of *α*-SMA^+^ blood vessels in the scar area of the cell-treated groups was increased significantly compared with that of the PBS-injection group (PBS-injection group v. two-injection group, ^∗∗^*P* < 0.01; PBS-injection group v. four-injection group, ^∗∗∗^*P* < 0.001). Significant difference could be found when comparing the four-injection group with the two-injection group, ^∗∗∗^*P* < 0.001.

Using anti-*α*-SMA antibody staining, we found that injections of hiPSC-MSC increased the number of *α*-SMA^+^ artery vessels in the scar area of the MSC-injection groups compared with that of the PBS-injection group (PBS-injection group: 6.12 ± 1.24 v. two-injection group: 8.625 ± 1.06, *P* < 0.05; PBS-injection group: 6.12 ± 1.24 v. four-injection group: 13.62 ± 2.19, *P* < 0.001). There was a significant difference when comparing two- and four-injection groups, *P* < 0.001 (Figure 8 and Table 6).

### 3.9. hiPSC-MSC Administrations Reduced Apoptotic Cell Number in the Border and Infracted Area

**Figure 9:**
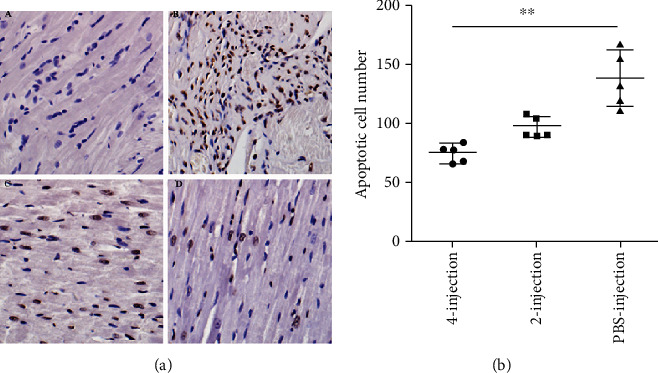
hiPSC-MSC administration reduced the apoptotic cell number in the infarcted and border area. (a) Detection of apoptotic cells by TUNEL staining. A Apoptotic cell was rarely found in sections of the sham operated rats. B Apoptotic cells in a section of the PBS-injection-treated rat. C Apoptotic cells in a section of the two-injection-treated rat. D Apoptotic cells in a section of the four-injection treated rat 400×. (b) The number of apoptotic cells in the infracted and border area of the cell-treated groups was reduced compared with that of the PBS-injection group, and there was a significant difference between the four-injection group and the PBS-injection group (^∗∗^*P* < 0.01).

Representative TUNEL staining images (cells with brown stain) of the four groups are shown, respectively, in Figure 9a. The results indicated that the cell number of apoptosis in the border and infracted area was reduced in the four- and two-injection groups (74.53 ± 7.42 and 97.33 ± 8 cells/hpf) compared with that of the PBS-injection control group (129.33 ± 33.61 cells/hpf), and significant reduction could be found in the comparison between the four-injection group and the PBS-injection group, ^∗∗^*P* < 0.01 (Figure 9b and Table 7).

## 4. Discussion

MSC infusions have been investigated as a treatment means for cardiac disease for more than a decade. Although accumulating evidence suggests that the MSC infusion can improve the cardiac function [34], however, information on the long-term dynamic outcomes of multiple hiPSC-MSC infusions on cardiac disease is unavailable.

The current study is based on the concept that a persistent, excessive inflammatory response contributes to progressive myocardial dysfunction post-AMI, and the chronic inflammatory response cannot be suppressed permanently by a single infusion of MSCs.

Human-induced pluripotent stem cells are an attractive stem cell source for cell therapy. Spontaneous mesoderm commitment of hiPSCs can be initiated by manually dissociation and followed by subsequent passaging or embryoid body formation. It usually takes more than 30 days to generate cells with increased MSC marker expression and bilineage mesenchymal differentiation potential from hiPSCs [27, 35–37]. Small molecules, such as SB431542 and VPA, play an impotent role in epithelial-to-mesenchymal transition induction [27, 31], and SB431542 is reported to be feasible in MSC derivation from hiPSCs, but it still takes more than twenty days to generate MSC-like cells [27].

Through modifying histone acetylation, VPA greatly accelerated the EMT process of hiPSC, and large number of MSC like cells could be obtained within 15 days in our research. Compared with the spontaneous commitment and SB431542 induction method, the VPA treatment method of obtaining MSC is effective and timesaving. The derived cells expressed MSC-related marker, secreted angiogenic and immune regulatory factors in levels comparable to that of UC-MSCs, except for HGF, and could be induced to experience chondrogenic and adipogenic differentiation, and more importantly did not form a tumor in vivo.

During the induction process, the expression of EMT-related genes N-cadherin and vimentin was upregulated to a very high level, and the expression of pluripotency-related genes Sox2 and Oct4 was downregulated or remained unchanged, indicating that VPA initiated the process of EMT through upregulating EMT-promoting genes and downregulating pluripotency-related gene expression. Although the EMT opposite process MET-related gene E-cadherin expression was upregulated for unspecified reasons, under the very high level expression of N-cadherin and vimentin, the upregulated E-cadherin expression could not prevent the initiation of EMT in the VPA-treated cells.

It has been reported that VPA might activate and stabilize neurons and astrocytes by stimulating the expression of trophic factors, and recent reports indicate that VPA treatment could enhance multipotency, migration, proliferation, and differentiation of MSCs [28, 29]. In our research, we found that low concentration (0.1-0.5 mM) of VPA could increase MSC viability, whereas concentrations that exceed 0.5 mM would inhibit cell proliferation and induce cell apoptosis in a dosage-dependent manner (data not shown) indicating its bifunctional property.

Since VPA initiate EMT through modifying histone acetylation (epigenetic alteration), we did not perform karyotype analysis on these hiPSC-induced MSCs, and tumor formation monitoring only lasted twelve weeks; hence, we do not know whether the injected cells would form tumor in a longer period.

Tissue origin greatly affects stem cell differentiation and function of the derived cells. The tissue-specific epigenetic profiles, such as DNA methylation (DNAm) patterns, may not be erased and reestablished completely during reprogramming of somatic cells into hiPSCs. This specific characteristic also remains upon redifferentiation into hiPSC-MSCs [38]. The low-level secretion of HGF in hiPSC-MSC may be related to the incomplete erasing of DNA methylation pattern during the reprogramming process.

Intravenous infusions of hiPSC-MSCs to rats were started one week after AMI induction and continued for the following two to six weeks, because evidence suggests that application of MSCs seven days after AMI is preferable to that given one day after AMI due to differences in the cardiac environment after AMI [16]. During the four rounds of infusions of 1 × 10^6^ hiPSC-MSCs to rats, no perceptible adverse effect and hiPSC-MSC-induced tumor formation were found over 12 weeks.

LVEF and LVFS are the most commonly used parameters for evaluating cardiac performance in clinical practice. LV functional deterioration following AMI remains incomplete after a few days [39]. LVEDD is a predictor of ventricular remodeling [40]. The pathophysiological changes of ventricular remodeling can be divided into early and late phases after AMI onset. The early phase involves expansion of the infarct area, and late remodeling involves time-dependent dilatation, distortion of ventricular shape, and mural hypertrophy [41].

We demonstrated that, after AMI induction, without intervention, the LVEF, LVFS, and LVEDD of the PBS-injection control group continued to deteriorate over 12 weeks. hiPSC-MSC treatment improved LVEF and LVFS and delayed the increase of LVEDD in the cell-treated groups. LVEDD increases among differently treated groups were insignificant within a 12-week period, perhaps due to ventricular remodeling being a chronic process.

The reasons for the poorer improvement of the cardiac function in the two-injection group may be because, without continuous provision of hiPSC-MSCs to model rats, the beneficial factors secreted by the hiPSC-MSCs were exhausted two to three weeks after the second cell injection; thus, the uncurbed inflammation continued for a period of time. With a short lifespan, the injected hiPSC-MSCs could not survive or multiply for more than three weeks [25, 26]. In addition, the infused heterogenic hiPSC-MSCs could have been cleared gradually by a system of phagocytosis, although it is known that heterologous MSCs can evade the immune system. This phenomenon emphasises the importance of multiple rounds of MSC infusions on sustaining cardiac function improvement.

Van Dijk and Sheu et al. showed that injections of adipose tissue-derived MSCs could promote angiogenesis and reduce the scar area significantly after AMI [16, 42]. In our experiment, the apoptotic cell number of the cell-treated groups was reduced; the total *α*-SMA^+^ artery number in the scar area in the cell-treated groups was increased, and the size of this area was shrunk significantly compared with that in the control group. These results are consistent with that of Van Dijk and Sheu et al., indicating that the increased angiogenesis played an important role in reducing the scar area and improving the cardiac function.

In conclusion, we demonstrated that MSC-like cells could be effectively induced from hiPSCs by simple VPA treatment, and multiple intravenous infusions of hiPSC-MSCs to AMI rats were an efficacious and safe measure, whereby to restore reduced cardiac function.

## Figures and Tables

**Table 1:** Primers used in quantitative real-time RT-PCR.

Genes	PCR primers
E-cadherin	Sense primer:5′-TTCTGCTGCTCTTGCTGTTT-3′ Antisense primer: 5′-TGGCTCAAGTCAAAGTCCTG-3′
N-cadherin	Sense primer: 5′-CCTGCGCGTGAAGGTTTGCC-3′ Antisense primer: 5′-CCAAGCCCCGCACCCACAA-3′
Vimentin	Sense primer: 5′-GCCCTTAAAGGAACCAATGA-3′ Antisense primer: 5′-AGCTTCAACGGCAAAGTTCT-3′
Oct3/4	Sense primer: 5′-GACAGGGGGAGGGGAGGAGCTAGG-3′ Antisense primer: 5′-CTTCCCTCCAACCAGTTGCCCCAAAC-3′
Sox2	Sense primer: 5′-GGGAAATGGGAGGGGTGCAAAAGAGG-3′ Antisense primer: 5′-TTGCGTGAGTGTGGATGGGATTGGTG-3′
GAPDH	Sense primer: 5′-CATCAATGGAAATCCCATCA-3′ Antisense primer: 5′-TTCTCCATGGTGGTGAAGAC-3′

**Table 2:** Statistical analysis of LVEF at the end of 12 weeks.

	LVEF	*P* value
PBS-injection	36.94 ± 8.31%	*P* < 0.001
Two-injection	57.9 ± 4.8%	
PBS-injection	36.94 ± 8.31%	*P* < 0.001
Four-injection	64.7 ± 2.4%	
Two-injection	57.9 ± 4.8%	*P* < 0.05
Four-injection	64.7 ± 2.4%	

**Table 3:** Statistical analysis of LVFS at the end of 12 weeks.

	LVFS	*P* value
PBS-injection Two-injection	19.75 ± 3.8% 30.67 ± 3.7%	*P* < 0.001
PBS-injection Four-injection	19.75 ± 3.8% 34.25 ± 5.4%	*P* < 0.001
Two-injection Four-injection	30.67 ± 3.7% 34.25 ± 5.4%	*P* > 0.05

**Table 4:** Statistical analysis of LVEDD at the end of 12 weeks.

	LVEDD(mm)	*P* value
PBS-injection Two-injection	9.36 ± 0.77 8.67 ± 0.86	*P* > 0.05
PBS-injection Four-injection	9.36 ± 0.77 8.02 ± 0.34	*P* > 0.05
Two-injection Four-injection	8.67 ± 0.86 8.02 ± 0.34	*P* > 0.05

**Table 5:** Statistical analysis of scar size at the end of 12 weeks.

	Scar size	*P* value
PBS-injection Two-injection	13.74 ± 3.4% 10.18 ± 2.16%	*P* > 0.05
PBS- injection Four- injection	13.74 ± 3.4% 7.08 ± 3.68%	*P* < 0.01
Two- injection Four- injection	10.18 ± 2.16% 7.08 ± 3.68%	*P* > 0.05

**Table 6:** Statistical analysis of angiogenesis at the end of 12 weeks.

	*α*-SMA^+^ artery number/hpf	*P* value
PBS-injection Two-injection	6.12 ± 1.24 8.625 ± 1.06	*P* < 0.05
PBS-injection Four-injection	6.12 ± 1.24 13.62 ± 2.19	*P* < 0.001
Two-injection Four-injection	8.625 ± 1.06 13.62 ± 2.19	*P* < 0.001

**Table 7:** Statistical analysis of cell apoptosis at the end of 12 weeks.

	Apoptotic cell number/hpf	*P* value
PBS-injection Two-injection	129.33 ± 33.61 97.33 ± 8	*P* > 0.05
PBS-injection Four-injection	129.33 ± 33.61 74.53 ± 7.42	*P* < 0.01
Two-injection Four-injection	97.33 ± 8 74.53 ± 7.42	*P* > 0.05

## Data Availability

All the data used to support the findings of this study are included within the article.
